# Supplementary material for: Low-Luminance Blue Light-Enhanced Phototoxicity in A2E-Laden RPE Cell Cultures and Rats
Source: Int J Mol Sci. 2019 Apr 11;20(7):1799. doi: 10.3390/ijms20071799 (PMC6480556; doi:10.3390/ijms20071799)
Supplement: Supplementary file 1 [file ijms-20-01799-s001.pdf]

SUPPLEMENTARY INFORMATION

Supplementary Table S1:

The Primer Sets of Genes Presented in This Study

HGNC Official Name (Official Symbol)[*1]	Accession No.[*2]	Sense / Antisense sequences [Product size (b.p.)]
Angiopoietin 1 (Ang-1)	NM_001146	GGGCACATTTGCACATACAG / GAAGGGAACCGAGCCTATTC [180]
Complement Factor H (CFH)	NM_000186	TTGCACACAAGATGGATGGT /GGATGCATCTGGGAGTAGGA [221]
Connective Tissue Growth Factor (CTGF)	NM_001901	CATCTTCGGTGGTACGGTGTA / CAGGCAGTTGGCTCTAATCATAG [291]
C-C motif chemokine ligand 2 (CCL-2)	NM_002982	CAGCCAGATGCAATCAATGC / GTGGTCCATGGAATCCTGAA [197]
Erythropoietin (EPO)	NM_000799	GTCCCAGACACCAAAGTTAA / AGGCCACTGACGGCTTTAT [184]
Glucose Transporter Member 1 (GLUT-1)	NM_006516	TCAATGCTGATGATGAACCTGCT / GGTGACACTTCACCCACATACA [163]
Homo sapiens hypoxia inducible factor 1 alpha subunit (HIF-1α)	NM_181054	GCTGGCCCCAGCCGCTGGAG / GAGTGCAGGGTCAGCACTAC [194]
Interleukin 1 beta (IL-1β)	NM_000576	TACGAATCTCCGACCACCACTAC / GTACAGGTGCATCGTGCACATAAGC [209]
Interleukin 12B (IL-12)	NM_002187	CACATTCCTACTTCTCCCTGAC / CTGAGGTCTTGTCCGTGAAG [92]
Matrix Metallopeptidase 9 (MMP-9)	NM_004994	GCGGAGATTGGGAACCAGCTGTA / GACGCGCCTGTGTACACCCACA [208]
Opticin (OPTC)	NM_014359	CTCTGCCCGTGCTGCCCAGT / GGCAAAGGCCCCGGGATAGA [156]
Kinase Insert Domain Receptor (VEGFR-2)	NM_002253	GCATGGTCTTCTGTGAAGCA / CCAGAGATTCCATGCCACTT [431]
Homo sapiens glyceraldehyde-3-phosphate dehydrogenase (GADPH)	NM_001289746	GGGTGTCGCTGTTGAA / CTGAGCTGAACGGGAAG [206]

[*1] The official human gene names/symbols are approved by HUGO Gene Nomenclature Committee (http://www.genenames.org/about/guidelines).

[*2] Accession number identified the sequence record for primer design is referred from NCBI GenBank.
